# Gastric Origin of Croup

**Published:** 1847-10

**Authors:** 


15. *Gastric Origen of Croup.*—Dr. Cain enters into a lengthened discussion to prove that "inflammation of the mucous membrane of the trachea and larynx, is sometimes caused by crudities in the stomach," adducing in support of his opinion in regard to croup, one case in which an attack supervened on a quantity of indigestable food having been taken into the stomach. He says Dr. Dickson, of South Carolina, in the only author, so far as he is aware, who has mentioned the connection of croup with the presence of indigestable substances. This opinion is so universally entertained by practitioners (although no written expression may have met Dr. C's eye), that no extended argument seems necessary to elucidate it. Dr. C's article is to be found in the Southern Journal of Medicine and Pharmacy. —*Wood's Quarterly Retrospect.*

